# Rethinking psychiatry with OMICS science in the age of personalized P5 medicine: ready for psychiatome?

**DOI:** 10.1186/1747-5341-8-4

**Published:** 2013-07-12

**Authors:** Nicola Luigi Bragazzi

**Affiliations:** 1 Department of Health Sciences (DISSAL), School of Public Health, University of Genoa, Via Pastore 1, 16132, Genoa, Italy; 2 DINOGMI, Department of Neuroscience, Rehabilitation, Ophthalmology, Genetics, Maternal and Child Health, Section of Psychiatry, University of Genoa, Genoa, Italy

**Keywords:** Biological psychiatry, Systems biology, Network medicine, Bioinformatics, New psychiatry, Personalized medicine, DSM-V, OMICS, Biologia psichiatrica, Biologia dei sistemi, Medicina delle reti, Bioinformatica, La nuova psichiatria, Medicina personalizzata, DSM-V, Scienze omiche

## Abstract

The Diagnostic and Statistical Manual of Mental Disorders (DSM) is universally acknowledged as the prominent reference textbook for the diagnosis and assessment of psychiatric diseases. However, since the publication of its first version in 1952, controversies have been raised concerning its reliability and validity and the need for other novel clinical tools has emerged. Currently the DSM is in its fourth edition and a new fifth edition is expected for release in 2013, in an intense intellectual debate and in a call for new proposals.

Since 1952, psychiatry has undergone many changes and is emerging as unique field in the medical area in which a novel approach is being demanded for properly treating patients: not the classical “one-size-fits-all” approach, but a more targeted and tailored diagnosis and therapeutics, taking into account the complex interactions among genes and their products, environment, culture and the psychological apparatus of the subject.

OMICS sciences, being based on high-throughput technologies, are systems biology related fields (like genomics, proteomics, transcriptomics and so on). In the frame of the P5 medicine (personalized, participatory, predictive, preventive, psycho-cognitive), they could establish links between psychiatric diseases, which are disorders with a final common symptomatology with vastly heterogeneous biological, environmental and sociological underpinnings, and by understanding the psychiatric diseases beyond their classic symptomatic or syndromal definitions using OMICS research, one can have a broader picture and unprecedented links and reclassification of psychiatric nosology. Importantly, by understanding the basis of heterogeneity in diseases through OMICS research, one could also personalize treatment of psychiatric illnesses.

In this manuscript, we discuss a gap in the current psychiatric research, namely the missing logical link among OMICS, personalized medicine and reclassification of diseases. Moreover, we explore the importance of incorporating OMICS-based quantitative dimensional criteria, besides the classical qualitative and categorical approach.

## Abstract (de)

**Additional non-english language abstract:**

La Psichiatria è andata incontro a numerosi cambiamenti, nel corso della sua storia. Il Manuale Diagnostico e Statistico delle Malattie Mentali (in inglese, DSM) è universalmente riconosciuto come il testo di riferimento per la classificazione e la diagnosi dei disordini psichiatrici, sebbene soffra di alcune limitazioni e fin dalla pubblicazione della sua prima versione nel 1952 sono stati sollevati dubbi sulla sua validità e affidabilità, così come è emerso il bisogno di altri e differenti strumenti metodologici di assessment psichiatrico. Attualmente il DSM è alla quarta edizione e la nuova quinta edizione è attesa per il 2013, in un intenso clima di dibattito intellettuale e in un aperto invito a contributi e proposte. Dal 1952 la psichiatria è emersa come campo unico all'interno delle discipline mediche in cui applicare un nuovo approccio per il miglior trattamento dei pazienti psichiatrici: un approccio non generalizzato e comune a tutti i malati, ma personalizzato e mirato alle specifiche esigenze di ognuno di essi. Tale approccio deve considerare la complessa rete di interazioni tra i geni e i loro prodotti, l'ambiente, la cultura e l'apparato psichico del paziente. Le scienze omiche (come la genomica, la proteomica, la transcrittomica e così via), essendo basate su biotecnologie che producono una enorme messe di dati, sono discipline che utilizzano un approccio sistemico e possono pertanto, nel contesto della medicina P5 (le 5 P stanno per personalizzata, predittiva, preventiva, partecipatoria, psico-cognitiva), mostrare relazioni mai immaginabili prima tra i geni e la psichiatria, offrendo così spunto a una possibilità di riclassificare le malattie psichiatriche. Queste sono disordini che hanno una sintomatologia in comune nonostante una ampia eterogeneità e hanno una notevole rilevanza sia biologica, che sociale e politica. Inoltre, comprendendo questa fitta rete di inter-relazioni, si potrà anche offrire un trattamento personalizzato mirato al singolo paziente. Col presente manoscritto, vogliamo individuare un gap nell'attuale ricerca in psichiatria, nello specifico il link tra le scienze omiche, la medicina personalizzata e la riclassificazione delle patologie psichiatriche. Inoltre, ci proponiamo di esplorare l'importanza di incorporare criteri classificativi basati sulle scienze omiche, ossia dati dimensionali e quantitativi, accanto all'approccio classico, qualitativo e categoriale.

## Introduction

Traditionally, psychiatric diseases have been considered as a cluster of symptoms (syndromes) and psychopathology has been the gold standard to make diagnosis. However psychiatric diseases are complex, multifaceted and multifactorial pathologies, characterized by high heterogeneity and variance and therefore classical methods have proven to be too simple or not completely adequate to capture this complexity [1].

The categorical approach, in fact, suffers from some drawback, like circular reasoning and ambiguity [2], in considering diseases such as static categories and discrete ontologies, separate one from the others. Moreover, it is very puzzling where to set the boundary between the “normal” (health status) and the “abnormal” (the disease) and this has not only academic and nosological issues, but above all social and political concerns [2].

Another disadvantage of using the categorical approach alone is the nosological overlap: under the same clinical umbrella, different diseases with different prognosis can co-exist. Categorical approach, being qualitative, should be complemented with a more fine-grained diagnostic tool. On the other hand, molecular classification can really help and improve the classical nosological taxonomy and thus ameliorate the outcome of patient management and care.

This aspect of integrated psychological and biological assessment, that is to say both quantitative and qualitative, categorical and dimensional, is to be stressed within the frame of personalized medicine (P5) and targeted therapeutics, which recently emerged as promising and exciting trends. P5 medicine (i.e., predictive, preventive, personalized, participatory and psycho-cognitive) [3-5] entails the addition of the psycho-cognitive domain as a conceptual evolution of P4 medicine, introduced by the molecular oncologist Leroy Hood [6]. P5 medicine, as advocated by Gorini, Pravettoni and Ozdemir, has abandoned the model of paternalism that characterized the 20^th^ century physician-patient interaction. Instead P5 is participatory and makes use of models and equations to predict patient's future health status in order to adopt the best strategy available (being predictive); is preventive in being proactive and not merely reactive; and is psycho-cognitive in that it adopts an integrated model in which psychological health is seen as a fundamental aspect of personal well-being. Psychology plays different roles in medicine: from the compliance and adherence to treatment, to the access of the treatment itself (traditional versus alternative or complementary) and ultimately to the therapeutic alliance of the broad meaning. The concept of disease is thus shifting from an atheoretical, context-free, “Platonic” model, to an approach that instead is more focused on patient's characteristics and needs [5].

While P5 medicine is biology-driven (personalized, predictive, preventive), it is also psychologically and ethically engaged (psychocognive, participatory). Therefore, it is really patient-centered.

**Figure 1:**
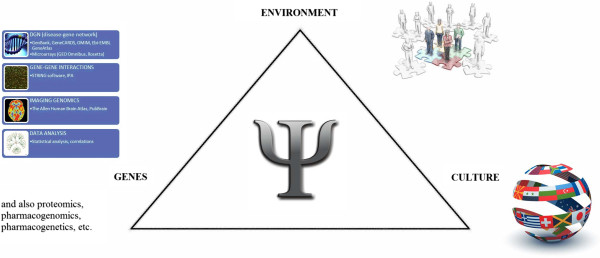
Triangle showing interactions among human genome, environment and culture in the pathogenesis of psychiatric diseases.

Thus, psychiatry is emerging as something of a unique medical field in which a novel approach is demanded and needed for properly treating patients through a more targeted and tailored diagnosis and therapy [1-14], taking into account the complex interactions among genes and their products, environment, and culture (as shown in Figure 1).

For this reason, both molecular (studied through novel biotechnologies such as gene microarrays and/or protein arrays) and psychodynamic aspects must be considered in order to fulfill the promises and true potential of a personalized psychiatry [15,16].

Moreover, since its very beginning, psychiatry has always had societal, ethical and political implications. The negative stereotypes, social stigma, and host of discriminating policies and discredit have hindered the process of psychiatric rehabilitation. P5 medicine, being multi-scalar and multi-dimensional, include the patient as an actor in the therapeutic process and incorporates “open knowledge production systems” in a truly holistic approach, may well capture the original concept of the individual person to acknowledge the dignity of the patient in the formulation and application of acts of care [17].

### From classical qualitative and categorical approaches to dimensional and integrated approaches

In this first section, we provide a brief overview of the historicity of the main approaches that have been proposed for a unitary psychiatric taxonomy, emphasizing the shift from a categorical qualitative approach, to a more refined dimension methodology.

### DSM/ICD model

The Diagnostic and Statistic Manual of Mental Disorders (DSM) was originally developed in 1952, upon the publication of the *DSM*-I. The DSM represents the first tool developed for *ad hoc* psychiatric diagnosis, even though its sources were implemented for epidemiological and social policy purposes, and not (at least initially) for the sake of diagnosis and treatment.

It is important to emphasize that DSM is a work in progress, and has undergone many changes through its evolution, shifting from a philosophical and phenomenological approach, to one that is more qualitative and psychometric, incorporating neo-Kraepelinian orientation (for an overview, the reader is referred to [2]).

The so-called Feighner criteria (and their expanded version, the Research Diagnostic Criteria, RDC, of the National Institute of Mental Health, NIMH, Psychobiology of Depression Collaborative Study, in 1978) have been acknowledged as the foundation of the version of DSM-III, which also incorporated the Robins-Guze criteria (i.e., describing five phases of psychiatric diagnostic formulation, namely clinical description, laboratory analysis, exclusion of other disorders, follow-up and family study) [18].

This same methodology has informed the International Statistical Classification of Diseases and Related Health Problems (ICD), developed by World Health Organization (WHO), while the Psycho-dynamic Diagnostic Manual (PDM) remains the psychoanalytical counterpart [19].

Although it was greatly improved throughout its different versions, problems persist in the DSM. Among the main limitations are the arbitrary threshold for diagnosis, the lack of quantitative criteria, potential abuse and/or misuse of the not otherwise specified (NOS) diagnostic category and overlap resulting from multi-axial structure (i.e., the “co-morbidity issue”).

As well, some empirical categories have disappeared from one version to the next, (e.g., homosexuality and some personality disorders), while others have been introduced (e.g., post-traumatic stress disorder, (PTSD)). Such categorical variation strongly suggests the need for more rigorous and evidence-based definition of diseases, as directly relevant to clinical diagnoses and treatment(s).

### Biopsychosocial model

The biopsychosocial model was first formally proposed by the psychiatrist George Engel [20] and has been incorporated - to greater or lesser extent - in the subsequent versions of the DSM. The model introduces distinctions within and between biological, psychological and social components, and advocates a more holistic orientation to disease, illness and medical care. However, post-Engelian interpretations of the biopsychosocial approach have tended to be somewhat obtuse [21-23]. Eric Kandel and Antonio Damasio have noted the linked and interwoven nature of a true biopsychosocial approach, such that psychiatric care does not belong to a separate and remote “psychological sphere” but also reflects and entails biological effects. In the same way, social components cannot be considered as completely distinct from biological and psychological domains.

### Meta-structure theory

Instead of a classification based on clinical presentation (as in DSM-IV, ICD10, and/or PDM), meta-structural taxonomy was proposed by Andrews and collaborators [24] as based on risk factors and intended as a more parsimonious classification. The proposed clusters are: neuro-cognitive disorders (cluster 1), neuro-developmental disorders (cluster 2), psychoses (cluster 3), emotional disorders (cluster 4), externalizing disorders (cluster 5) [24]. The proposed axes are only two and currently two variants exist.

Yet, this approach suffers from many of the same limitations as the previously described orientations.

### From categorical approaches to dimensional taxonomies: Endophenotype theory

An endophenotypic approach represensts a considerable step further and it is intriguing to note that this model although proposed for the psychiatric diseases, also may be extended to other diseases (e.g., diabetes, cancer, metabolic pathologies, etc.).

The endophenotype makes use of biological markers, described using the Buchsbaum criteria (i.e., a biomarker or a biological trait as a measurable indicator of a disease, which may be or may be not casual) and the Gottesman-Gould criteria for diagnosing an endophenotypic trait, which are that 1) the endophenotype is associated with illness in the population; 2) the endophenotype is heritable; 3) the endophenotype is disease state-independent; and 4) endophenotype and illness co-segregate within families [25].

This is fundamental to the suggested changes we propose to inform the use of the new DSM-V.

### Insights from systems biology, neuroinformatics and OMICS sciences

We posit that the traditional conception of “homeostasis” and the difficulty of distinguishing “normal” and an “abnormal” status can be compensated – if not resolved to great extent – by the idea of a personalized inner dynamical equilibrium that exist among the genetic and biological components, developed and expressed psychological resources traits and expressions, and the environment. Baselines and threshold values for biological (and, by effect psycho-social) system sensitivities and responses vary from person to person, as well in the same person throughout the lifespan. Thus the shift from a healthy status to a pathological status is often not a binary condition, but rather reflects a continuum, wherein disease emerges as a transformation of the properties of system(s) that have gradually become less adaptive [26-28].

Not merely focusing on single pieces of the proverbial puzzle, but instead taking a more comprehensive view of the picture that entails the systems involved, with and through approaches from systems biology, neuroinformatics and OMICS-based sciences to be implemented in psychiatric assessment and treatment [26].

According to etymology, OMICS is derived from the Sanskrit OM, which means “completeness and fullness”, and thus, a holistic, systems-oriented approach [29], which may be well-positioned to fill the gap between the need for a rigorous and rational psychiatry and the need for a personalized medicine.

### The “Psychiatome”

The interplay of genomics, proteomics, transcriptomics, toponomics, metabolomics and neuroimaging is emerging as powerful tool to analyze psychiatric disorders and providing patients personalized care. Barabási introduced the concept of “diseasome” [30] in the context of network medicine. Herein, we investigate the putative relationships among and between biological and environmental factors in psychiatric diseases, in what we call “*psychiatome*”, inspired by the concept of “diseasome”.

Our approach is to assess the shared genes (via mining through genetic databanks), shared networks (using inferred proteomics data) and shared networks of anatomical regions (i.e. toponomics, exploiting the PubBrain tool) in such a way as to develop a unique picture of salient inter-relationships that may subserve or be reflected by psychiatric disorders.

In attempting re-construct the complex genetic architecture of the psychiatric disorders (as multi-factorial diseases), we take into account whole-genome association (*WGA*) studies, copy-numer variant (*CNV*), pathways-based analysis (*PBA*), micro-arrays-based studies, single nucleotide polymorphisms (*SNPs*) studies, quantitative trait, loci and allelic heterogeneity studies.

**Figure 2:**
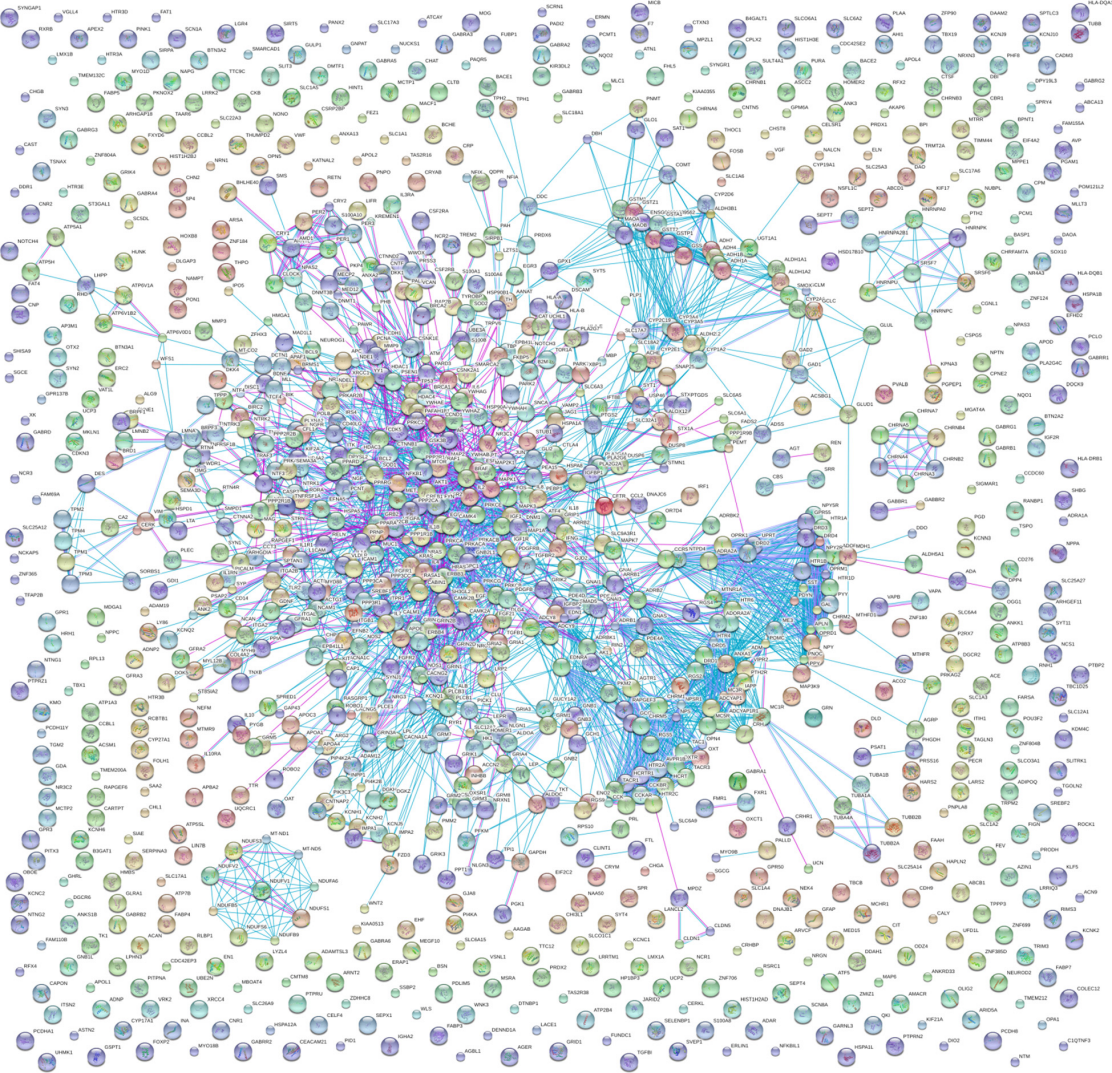
**Gene**-**gene connections of all genes involved in psychiatric diseases.** Edges between nodes represent the known molecular interactions.

**Figure 3:**
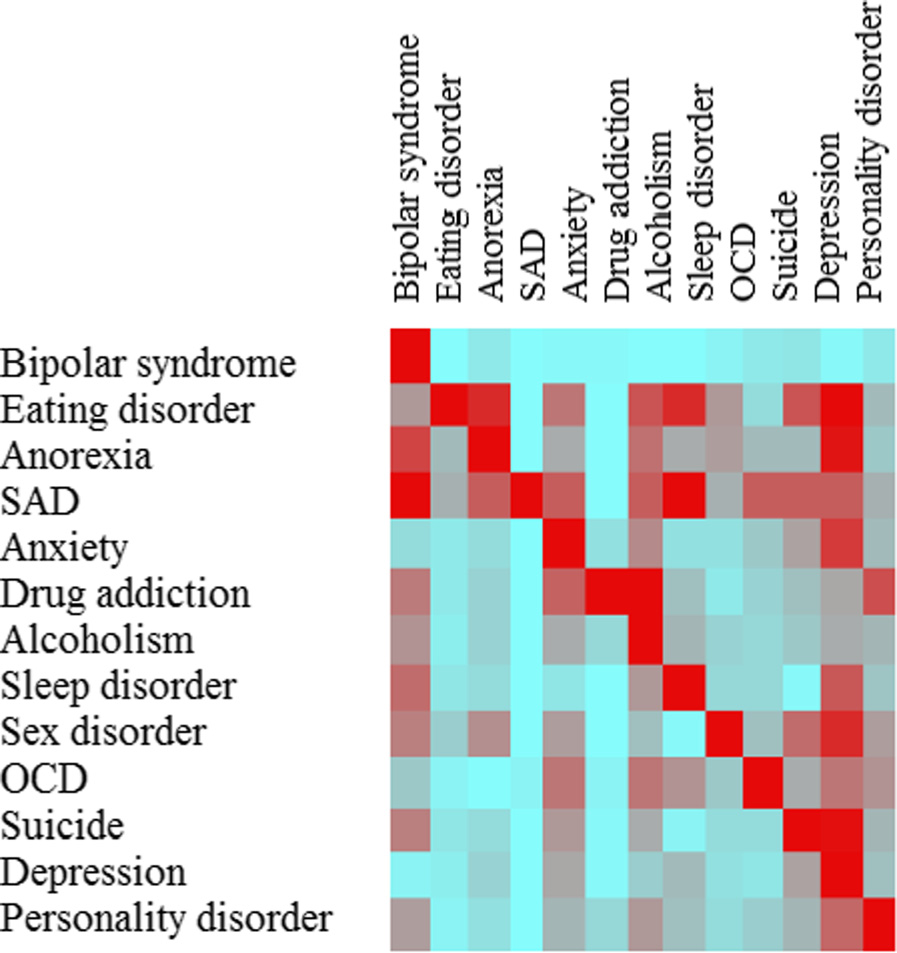
**Heat-map of psychiatric disease (where SAD stays for seasonal affective disorder).** The color bar indicates the degree of genomic overlap between a psychiatric disease and another (in terms of commonly shared genes), being represented in light blue if weaker and in red if stronger.

**Figure 4:**
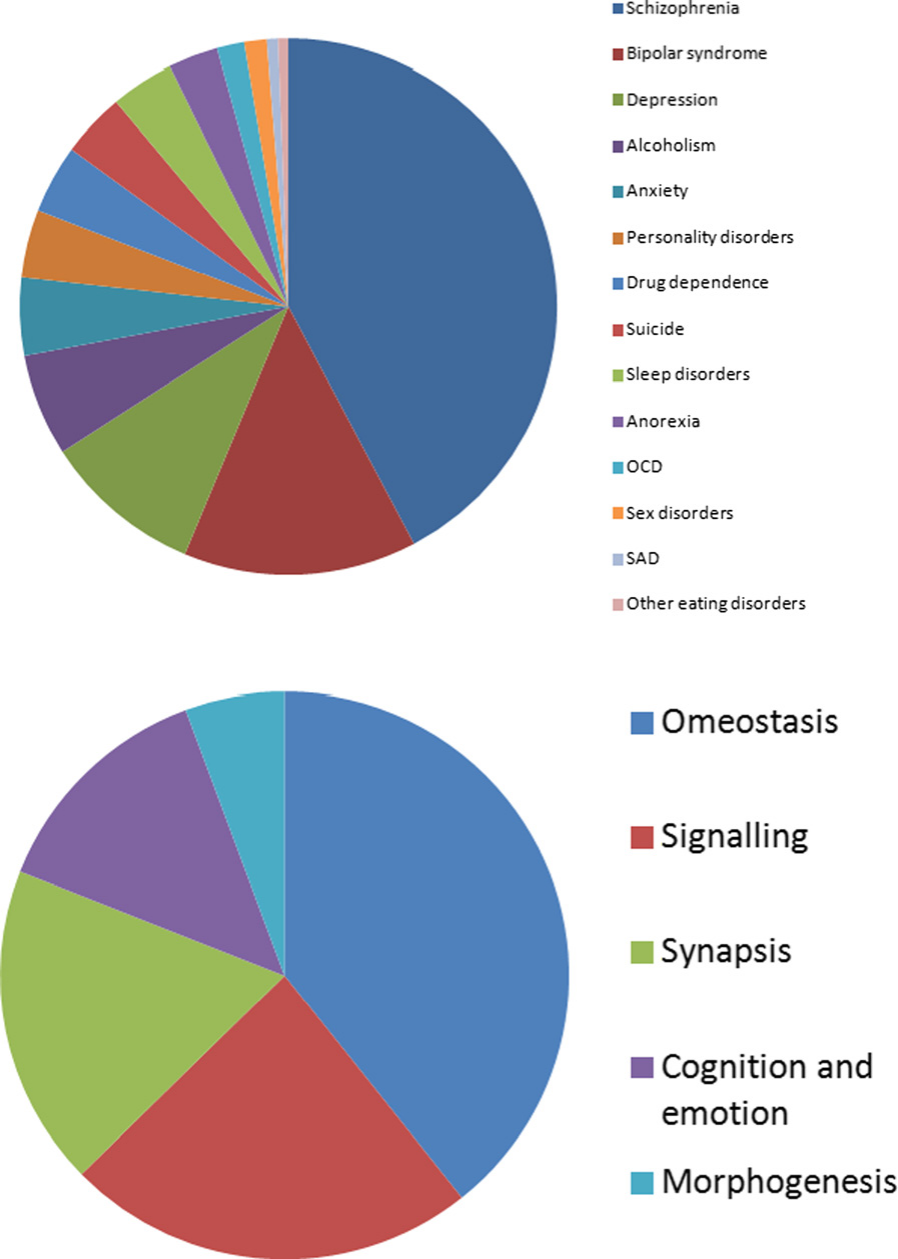
**Percentages of genes found for each disease after mining different databases (top) and GO (Gene Onthology) functional enrichment analysis (bottom).** GO, being a set of semantic associations from biological concepts to specific genes, enables scientists to make inferences about a particular list/cluster of genes, from a systems biology perspective, rather than focusing on a single gene.

Other complex diseases (e.g., heart diseases, diabetes, asthma, hypertension) are in fact characterized by the involvement of different genes. The possible connections among different genes involved in our proposed psychiatome schema are shown in Figure 2, and the heat-map matrix depicting relationships among the different psychiatric diseases is represented in Figure 3, with their respective gene onthologic (GO) functional enrichment in Figure 4. Delineating common shared-genes and genomics-relationships among and between psychiatric diseases can be useful in shaping the concept of “spectrum” disorder (as merely a static binary notion of “disease”) and preparation for use of the new DSM-V in practice [31].

But a truly personalized psychiatry is a broader concept than simply of pharmacogenomics, genotyping or other molecular investigations: it also includes the cultural and spiritual beliefs and practices of the patient [32]. This is particularly true also because from an evolutionary point of view, culture and genes have co-evolved such that culture can influence human genome [33-35], just as the expression of traits and actions arising from the genome can affect culture [36], as pictorially shown in Figure 1.

## Discussion

In this light, we believe that there is urgent need for a more comprehensive personalized psychiatric medicine [37]. Moving from the well-known epidemiological triangle (Figure 1), we have postulated the necessity of considering psychiatric disease from multiple points of view, given that psychiatric disorders are the result of complex gene-environment-culture interactions and we have over-viewed some tools doctors can exploit.

Psychiatric diseases tend to be interrelated (Figures 2 and 3), and exert distinct molecular signatures, and behavioral markers [38].

Molecular and genomic algorithms [39-41], nanobiotechnologies [42-45], OMICS-derived data and clinical tests and questionnaires [46] can all be useful to unravel the ambiguities of psychiatric pathogenesis if – and only if - combined and integrated.

Classical qualitative approaches have limitations on both theoretical and pragmatic grounds. Considering a more dimensional methodology, could overcome many of these limitations and thereby be instrumental to improving psychiatric diagnoses and treatments. OMICS sciences, based on high-throughput technologies, are systems biology related fields, which in a framework of P5 medicine could help to establish critical links between psychiatric diseases, (as disorders with a final common symptomatology with vastly heterogeneous biological, environmental and sociological underpinnings), and could thereby afford an understanding of psychiatric disease beyond classic symptomatic or syndromal definitions, perhaps leading to better interpretation and use of psychiatric nosology. Perhaps most importantly, by fostering such understanding through OMICS research, could also enable a meaningfully personalization of psychiatric care.

## Conclusion

In this paper, we offer but an early and limited view to the potential benefits of OMIC-science in, and for psychiatry. Further studies must be undertaken to better characterize the relationships between genes, culture, and environments, but we hold that a P5-based psychiatry can uphold these tasks.

We have addressed what we believe to be a gap in current psychiatric research and practice, namely, the missing link between OMICS, reclassification of psychiatric disorder and personalized medicine. In posing the importance of incorporating OMICS-based quantitative dimensional criteria, (supplemental to the classical qualitative and categorical approaches), we assert that steps toward a new psychiatry should include:

1) incorporating OMICS-based data to diagnostic criteria, taking biological and OMICS-derived markers not as external validators, but as intrinsic components of assessment;

2) shift emphasis from reactive, *post*-*hoc* assessments, to more quantitative diagnostic and prognostically predictive approaches;

3) provide bio-psychosocial (and culture-spiritual) personalized diagnoses and treatments.

Perhaps the “psychiatome” will provide an adequate translational framework for both psychiatric research and practice, being holistic and broad, rather than narrow and simplistic, even though this promising paradigm at present is still at an early stage of its development and implementation.

## Competing interest

The author declares that they have no competing interests.
